# Broadband Perfect Absorber with Monolayer MoS_2_ and Hexagonal Titanium Nitride Nano-disk Array

**DOI:** 10.1186/s11671-017-2232-4

**Published:** 2017-07-25

**Authors:** Dewang Huo, Jingwen Zhang, Hao Wang, Xiaoxuan Ren, Chao Wang, Hang Su, Hua Zhao

**Affiliations:** 1 Institute of Modern Optics, Department of Physics, Harbin Institute of Technology, Harbin, 150001 China; 2 Key Laboratory of Micro-Optics and Photonics Technology of Heilongjiang Province, Harbin, 150001 China

**Keywords:** Metamaterial absorber, Finite difference time domain, Titanium nitride, Hexagonal nano-disk array, Monolayer MoS_2_

## Abstract

A broadband metamaterial absorber (MA) composed of hexagonal-arranged single-sized titanium nitride (TiN) nano-disk array and monolayer molybdenum disulfide (MoS_2_) is studied using finite-difference time-domain (FDTD) simulations. The structure of TiN nano-disk array/dielectric silica (SiO_2_)/aluminum (Al) is adopted in our design. By optimizing the dimension parameters of the structure, an average absorption of 96.1% is achieved from 400 to 850 nm. In addition, by inserting a monolayer MoS_2_ which has high absorption at the short wavelength side underneath the TiN nano-disk array, an average absorption of 98.1% over the entire visible regime from 400 to 850 nm was achieved, with a peak absorption near 100% and absorption over 99% from 475 to 772 nm. Moreover, the absorber presented in this paper is polarization insensitive. This compact and unique design with TiN nano-disk/monolayer MoS_2_/ SiO_2_/Al structure may have great potential for applications in photovoltaics and light trapping.

## Background

Metamaterials are able to tailor the amplitude, phase, and polarization responses of the incident light in an unprecedented way. In particular, absorption enhancement with metamaterials is one of the most interesting topics associated artificially engineered metamaterials [1–5]. Several metamaterial structures were demonstrated as high-performance light absorbers, such as dense nanorods and nanotubes [6, 7], multilayer planar photonic structures [8–10], and photonic crystals [11]. In the past decade, Au [12] and Ag have been intensively investigated [13–16] for designing absorbers. At earlier stage, most research activities have focused on the absorption of the electromagnetic field within a narrow waveband with structures of metal nanoparticles, periodic gratings, and metal/dielectric/metal thin layers [17–20]. However, broadband absorption throughout the entire visible regime is important for photovoltaic and thermo-photovoltaic cells. Driven by real needs in realistic applications, research works on broadband absorption were reported in recent years. The absorber with a nanostructured top silver film composed of crossed trapezoidal arrays offers broadband and polarization-independent resonant light absorption with an average measured absorption of 0.71 against a simulated absorption of 0.85 over the entire visible regime (400–700 nm) [16]. The broadband absorption was further improved with an absorber based on multiple metal/dielectric/ metal layers with an average simulated absorption of 93% over the entire visible region [14]. In order to get better broadband absorption, semiconductor-based oxides and transition metal nitrides [21, 22] were proposed as alternative plasmonic materials. Specifically, transition-metal nitrides such as TiN or ZrN can serve as substitutes for conventional noble metals in the visible waveband [21]. A broadband metamaterial absorber based on TiN with square ring array shows an average absorption of 95% over the entire visible regime (400–800 nm) [23]. And absorption over 98% from 560 to 675 nm was obtained in a broadband metamaterial absorber with TiN and indium tin oxide transparent conducting films, while the average absorption was less than 85% for the short waves from 400 nm to 500 nm [24]. Recently, monolayer MoS_2_ shows great potential for generating various optoelectronic devices [25–34] and for photocatalytic applications owing to high absorption at the short wavelength side [35, 36]. The broadband absorber with metal Ag metasurface and a monolayer MoS_2_ was studied yet with average absorption less than 90% [37]. In this work, a more compact absorber with a monolayer MoS_2_ and hexagonal-arranged TiN nano-disk array is proposed, with as high as 98.1% average absorption over the entire visible region extending to near-IR (from 400 to 850 nm). This structure should be promising for photovoltaic applications.

## Methods

**Fig. 1:**
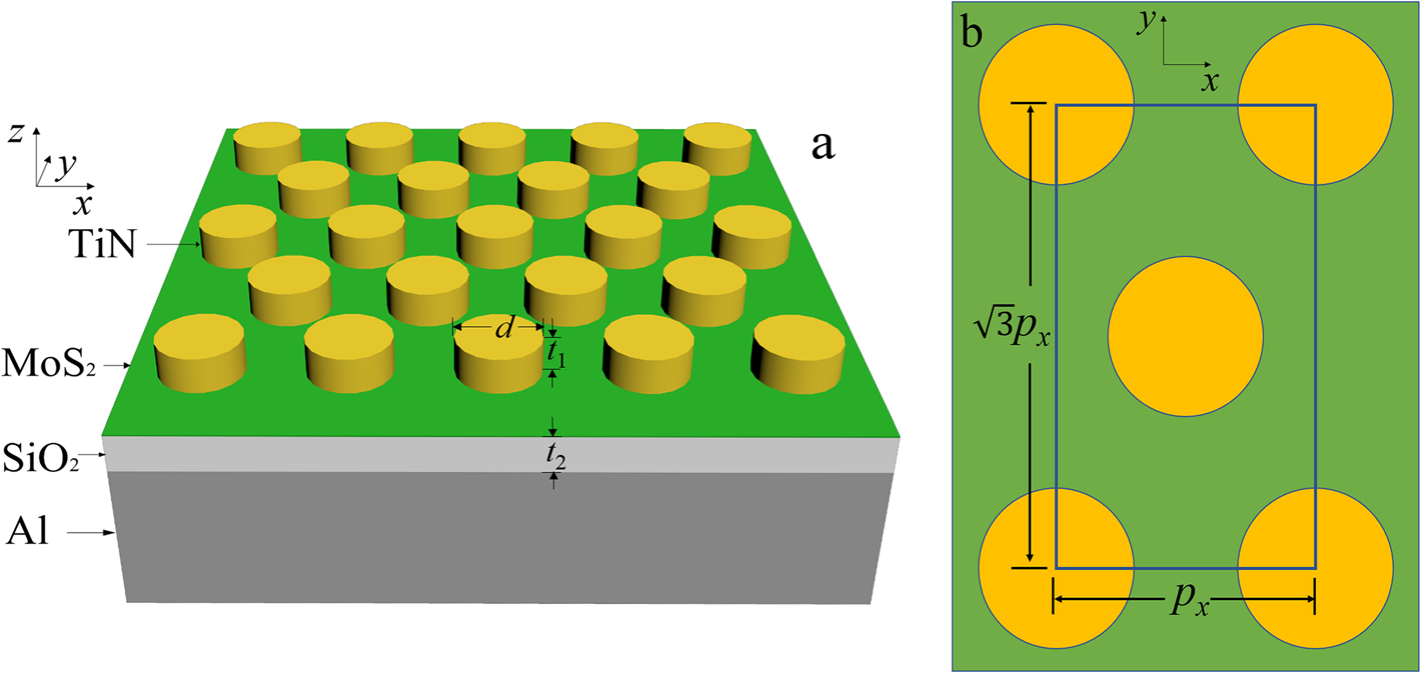
**a** Schematic of the proposed TiN nano-disks/monolayer MoS_2_/SiO_2_/Al structure. **b** Top view of a rectangle unit cell

The initial structure of our absorber and the top view of a unit cell are schematically shown in Fig. 1. A layer of dielectric SiO_2_ is sandwiched between a TiN nano-disk array and an aluminum (Al) substrate. The single-sized TiN nano-disks are hexagonally arrayed upon the SiO_2_ film with the same pitch. A monolayer MoS_2_ of thickness 0.625 nm is inserted underneath the nano-disk array. The structure parameters are denoted as follows: *p*_*x*_ and *p*_*y*_ = $\sqrt{3}px$ are the periodic lengths of the rectangle unit cell along the *x*- and *y*-directions, respectively; *d* is the diameter of the TiN nano-disk; *t*_1_ and *t*_2_ are the thicknesses of the top TiN nano-disk and the SiO_2_ film, respectively. An aluminum film is chosen as the substrate with thickness 500 nm, far thicker than the light penetration depth in the spectral range we studied.

The finite-difference time-domain (FDTD) method was employed in the simulation with the software package from Lumerical FDTD Solutions. The light is assumed incident normally on the absorber towards the −*z*-direction. In this simulation, the *z*-direction has two perfectly matched layers, and the periodic boundary conditions are set in the *x*- and *y*-directions. The absorbance can be calculated from the corresponding transmittance (R) and transmittance (T) with *A* = 1−*R*−*T*. It is easy to see the transmittance is always zero in our case because the Al substrate is far thicker than the light penetration depth in the spectral range and serves as a mirror in forming a resonance cavity with the nano-disk array to enhance the absorption. In the simulations, non-uniform mesh sizes were used in regard to different layer sizes and the specific settings were as following: a mesh size of 2.0 nm × 2.0 nm × 0.1 nm was employed in the monolayer MoS_2_; a mesh size of 2.0 nm × 2.0 nm × 2.0 nm was set in other simulating regions.

**Fig. 2:**
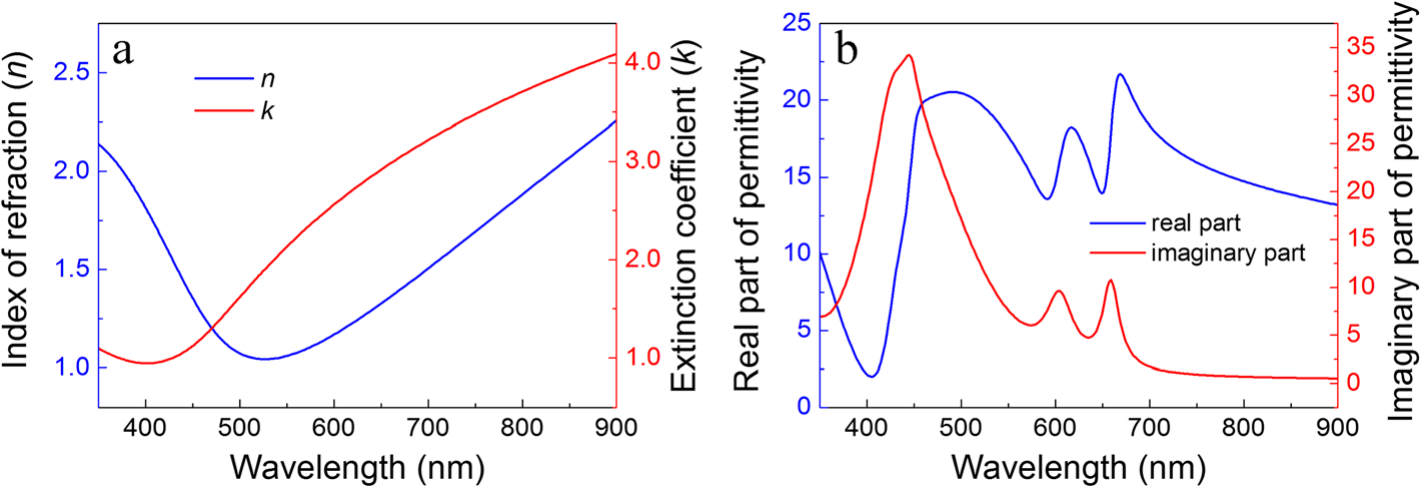
**a** Dispersion of TiN layer: *n* is the refractive index and *k* extinction coefficient. **b** Dispersion of monolayer MoS_2_

The refractive index curve of SiO_2_ spacer layer was adopted from the material base of the software Lumerical FDTD Solutions. The related material parameters of TiN were borrowed from Ref. [38], and the dispersion curve of the monolayer MoS_2_ was obtained from Ref. [39]. The fitted dispersion curves of TiN and monolayer MoS_2_ are exhibited in Fig. 2. In the visible regime, the TiN is proposed to replace the noble metal like Au or Ag in realizing the excitation of LSPR [21], since the TiN shows much higher extinction coefficient compared to the noble metals. However, relatively low extinction coefficient at the shortwave edge indicates unsatisfying absorption performance. Fortunately, a monolayer MoS_2_ possesses quite high extinction coefficients, especially at the short wave side; it can be introduced to the TiN nano-disk/SiO_2_/Al structure to improve broadband absorption over the entire visible regime. In addition, the monolayer MoS_2_ is of direct-gap semiconductor in which the electrons can be easily excited. And with decent thermoelectric property [40], it would make good use of the energy absorbed by the proposed structure and benefit for the solar energy applications.

## Results and Discussion

The absorption performance of the TiN nano-disk/SiO_2_/Al structure is studied firstly. In order to optimize the performance of the structure, the dependences of the absorption spectra on the diameter and the thickness of TiN nano-disks, and the thickness of SiO_2_ spacer layer, respectively, have been studied with *x*-polarized incident light with the optimized period *p*_*x*_ at 200 nm.

**Fig.3:**
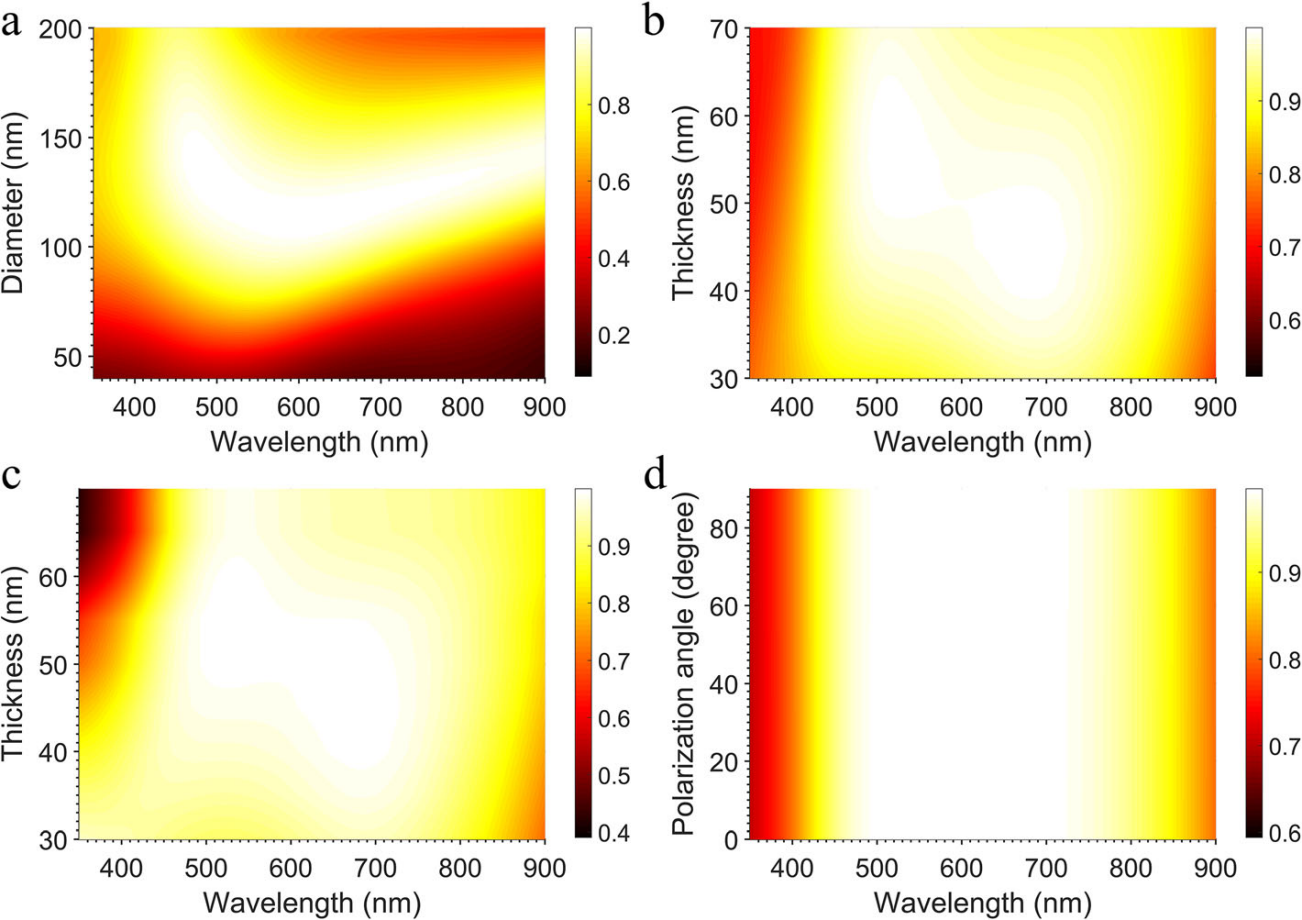
**a** Absorption spectra versus diameter of the top TiN nano-disk with the parameters fixed at *p*_*x*_ = 200 nm, and *t*_1_ = *t*_2_ = 50 nm. **b** Absorption spectra versus thickness of the top TiN nano-disks with *p*_*x*_ = 200 nm, *d* = 120 nm, and *t*_2_ = 50 nm. **c** Absorption spectra versus thickness of the SiO_2_ layer with *p*_*x*_ = 200 nm, *d* = 120 nm, and *t*_1_ = 50 nm. **d** Spectra versus polarization angle of the incident light with parameters set as *p*_*x*_ = 200 nm, *d* = 120 nm, and *t*_1_ = *t*_2_ = 50 nm. The *color bar* denotes the absorption value

Since the electric and magnetic fields in the unit cells are strongly influenced by the dimensions of the absorber [28, 41], the absorption spectra with the different diameters of TiN nano-disks were studied. Figure 3a shows the absorption spectra versus diameters of the top TiN nano-disks for *p*_*x*_ = 200 nm, and *t*_1_ = *t*_2_ = 50 nm. The resonance absorption increases when the diameter of the TiN nano-disks increases from 40 to 120 nm, then the absorption decreases with the diameter gradually approaching to 200 nm. Structure proposed possesses the best absorption performance over the visible regime when the diameter is around 120 nm.

For the same reason, the absorption dependence on the thicknesses of TiN nano-disks was also investigated. Figure 3b shows the absorption spectra versus the thickness of the top TiN nano-disks when other parameters were fixed at *p*_*x*_ = 200 nm, *d* = 120 nm, and *t*_2_ = 50 nm. It is apparent that the resonance absorption peak wavelength has a redshift while the *t*_1_ increases, and the resonance absorption bandwidth becomes wider from *t*_1_ = 30 to 50 nm. As a result, for *t*_1_ = 50 nm, the best absorption performance is achieved with wavelengths ranging from 453 to 797 nm, which is about 350 nm wide, with the absorption higher than 95%.

Moreover, the thickness of the SiO_2_ spacer layer is also a crucial parameter for determining magnetic resonance of the structure. From the absorption spectra versus thickness of SiO_2_ spacer layer in Fig. 3c, it is seen that the central wavelength of the resonance absorption peak is redshifted with increasing thickness of SiO_2_, and the optimized thickness is *t*_2_ = 50 nm while the rest parameters were set at *p*_*x*_ = 200 nm, *d* = 120 nm, *t*_1_ = 50 nm. One can see that the TiN nano-disk/SiO_2_/Al structure offers a quite satisfying broadband absorption with an average absorption of 96.1% from 400 to 850 nm.

**Fig. 4:**
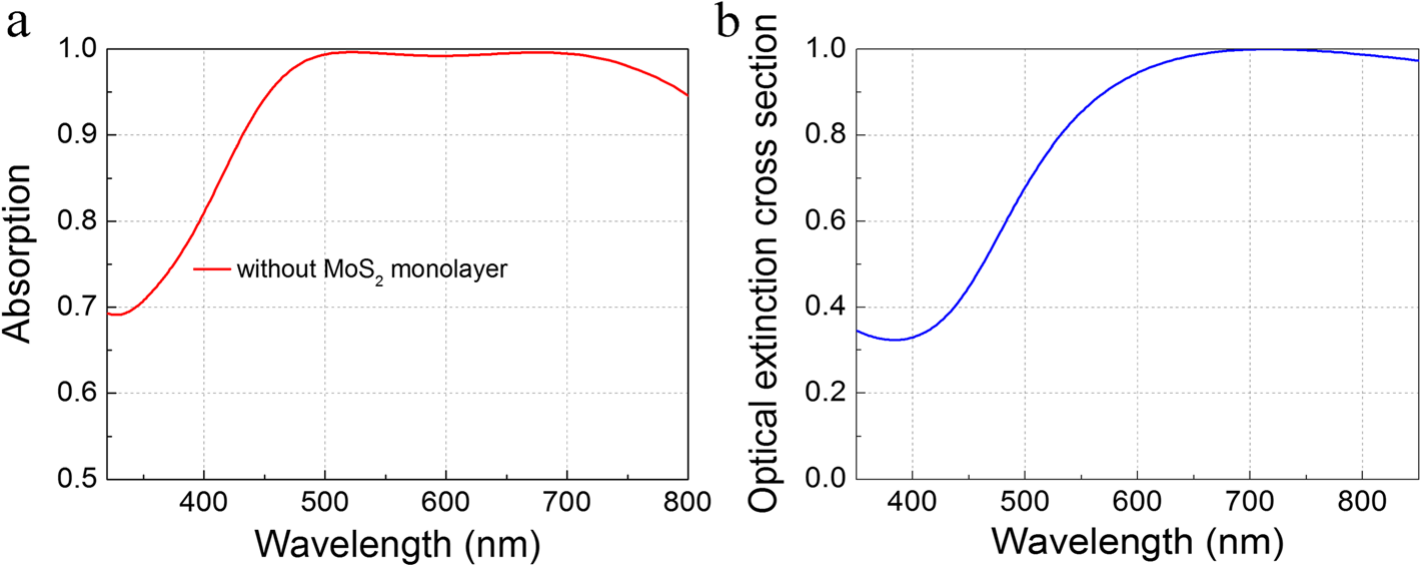
**a** Absorption spectra without monolayer MoS_2_ for *p*_*x*_ = 200 nm, *d* = 120 nm, and *t*_1_ = *t*_2_ = 50 nm. **b** Normalized optical extinction cross section of TiN nano-disk illuminated with plane wave at normal incidence

To understand the mechanism behind the absorption peak around 680 nm in Fig. 4a, the coupled dipole approximation approach was employed by treating a nano-disk as a polarizable dipole. Since the size of a TiN nano-disk is far smaller than the wavelength of visible light, the quasi-static approximation is valid in dealing with the case. In the quasi-static approximation, each nano-disk illuminated with incident light could be treated as a dipole with polarizability [42],

1 $$ \alpha \propto V\frac{\varepsilon_1-{\varepsilon}_2}{\varepsilon_2+L\left({\varepsilon}_1-{\varepsilon}_2\right)} $$

Here, *V* denotes the volume of TiN disk, *ε*_1_ = *ε*_1*r*_ + *ε*_1*i*_ is the frequency-dependent dielectric permittivity of TiN nano-disk, and *ε*_2_ is the dielectric constant of embedding medium SiO_2_. When the applied electric field of the incident light is polarized parallel to the disk (i.e., *xy* plane), the shape factor can be written as [42]

2 $$ L=\frac{g}{2{e}^2}\left(\frac{\pi }{2}-ta{\mathrm{n}}^{-1}g\right)-\frac{g^2}{2} $$

3 $$ g=\sqrt{\frac{1-{e}^2}{e^2}} $$

4 $$ {e}^2=1-\frac{4{t}_1^2}{d^2} $$

Here, *d* is the diameter of the TiN nano-disk and *t*_1_ is the thickness of the TiN nano-disk. Thus, the optical extinction cross section *σ*_ext_can be obtained from the polarizability [12, 43]

5 $$ {\sigma}_{ext}\propto k\operatorname{Im}\left(\alpha \right) $$

As aforementioned, the quasi-static approximation is applicable in calculating the optical extinction cross section of a single TiN nano-disk. The normalized optical extinction cross section of the nano-disk is shown in Fig. 4b, which has similar trend to the spectrum in Fig.4a with *p*_*x*_ = 200 nm, *d* = 120 nm, *t*_1_ = 50 nm, and *t*_2_ = 50 nm. The corresponding wavelength for the maximum optical extinction cross section is about 715 nm, close to the peak wavelength around 680 nm of the absorption spectrum from the simulation result. Indeed, the numerical result is not completely consistent with the absorption spectrum, because we only took the dimensions of TiN nano-disk into consideration to simulate the LSPR absorption peak but ignored the cross talks between the nano-disks and the magnetic resonance in the gap which should have significant influence on broadening the perfect absorption band and contribute to the improvement of the absorption performance in our structure. This will be explained in the following sections.

**Fig. 5:**
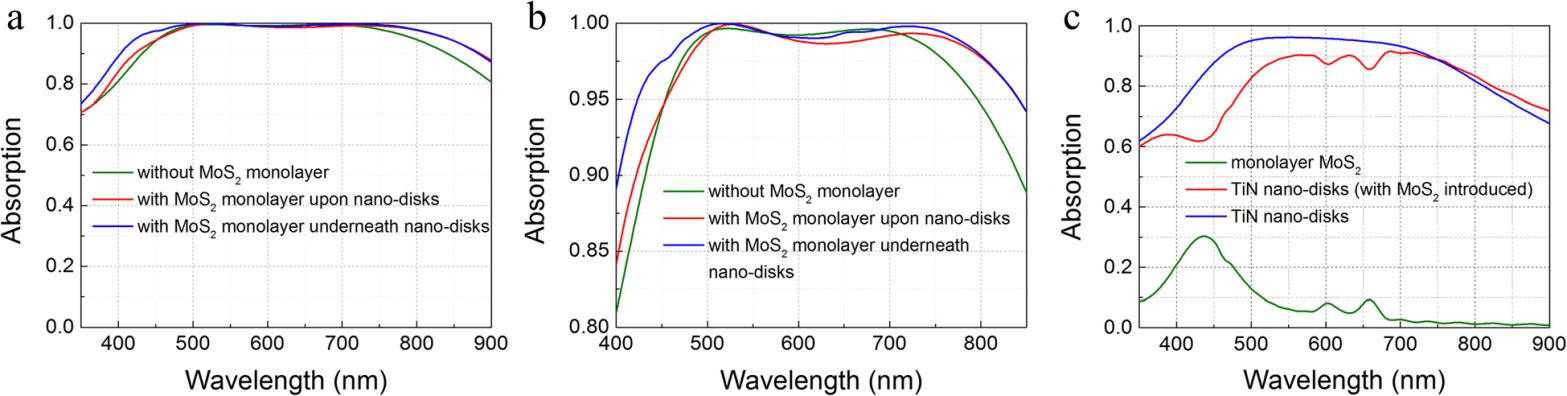
The absorption spectra **a** and the enlarged absorption spectra **b** of the cases that the monolayer MoS_2_ is introduced underneath TiN nano-disk array, upon the TiN nano-disks and not introduced, respectively. **c** The absorption of the TiN nano-disks and monolayer MoS_2_

**Fig. 6:**
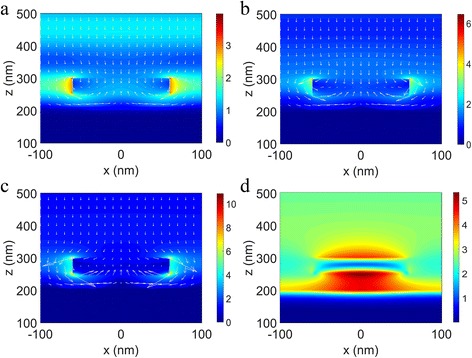
The distributions of the electric field *|* ***E*** *|* and the Poynting vectors in the *xz* plane at *y* = 0 of a unit cell illuminated with light at wavelengths *λ* = 402 nm (**a**), 502 nm (**b**), and 680 nm (**c**), as well as the magnetic field distribution

To raise absorption at the short wavelength edge, a monolayer MoS_2_ is introduced into the TiN nano-disk/SiO_2_/Al structure as shown in Fig. 1a, by inserting it upon and underneath the nano-disk array, respectively. The parameters were set as *p*_*x*_ = 200 nm, *d* = 120 nm, *t*_1_ = 50 nm, and *t*_2_ = 50 nm based on optimized results obtained previously. The electric field around the nano-disks is enhanced due to the excitation of LSPR as shown in Fig. 6. Consequently, the enhanced electric field strengthens the absorption in ultrathin monolayer MoS_2_, resulting in better absorption performance for the both cases as shown in Fig. 5a, b. Without MoS_2_ in the TiN nano-disk/SiO_2_/Al structure, the best absorption performance is obtained with a peak absorption near 100% and average absorption of 96.1% from 400 to 850 nm. With the monolayer MoS_2_ inserted underneath the TiN nano-disk array, a peak absorption near 100% is also observed. Compared to the case without monolayer MoS_2_, the band of absorption over 95% is broadened about 80 nm ranging from 424 to 842 nm, and the absorption of light wavelength at 400 nm is increased from 81 to 89%. As a result, the average absorption from 400 to 850 nm is improved from 96.1 to 98.1% with an about 300-nm wide wavelength range for the near 100% absorption from 475 to 772 nm. With MoS_2_ layer upon the nano-disk array, the total performance is also improved with averaged absorption of 96.8% from 400 to 850 nm. From the discussion above, it is seen that inserting monolayer MoS_2_ underneath the nano-disk array performs better in improving the absorption performance the structure proposed. To clarify the contribution of the monolayer MoS_2_ to the total structure, the absorptions of monolayer MoS_2_ and TiN nano-disks were calculated and shown in Fig. 5c. After introducing a monolayer MoS_2_, the absorption of the nano-disks at short wavelength edge slightly decreases. However, due to high absorption in monolayer MoS_2_, the total absorption is still increased at short wavelength edge of the spectra. At the long wavelength edge, the absorption by the nano-disks gets higher with introducing monolayer MoS_2_. As a whole, the absorption bandwidth is widened with the monolayer MoS_2_ underneath the TiN nano-disks.

Furthermore, the influence of polarization angle of incident light has also been studied. Figure 3d shows that the absorption spectra are barely influenced by the polarization angle of the incident light, as reported in some other metamaterial design [44–46]. The rotational symmetry of circular nano-disk ensures no difference with varying polarization angle at normal incidence. Additionally, the hexagonal array has a threefold rotational symmetry which makes the absorption insensitive to the polarization angle at normal incidence [44–47]. As a result, the total absorption in the structure is polarization insensitive.

To see how light is absorbed in MA structure, the field distributions and Poynting vectors which represent the energy flows for different resonant wavelengths are studied. In Fig. 6a–c, the electric field distributions are plotted on a cross section through the *xz* plane at *y* = 0, which indicate that the LSPR occurs to enhance the electromagnetic field around the nano-disk and confined the electromagnetic field among nano-disks in all the three cases corresponding to wavelengths 402, 502, and 680 nm, respectively. The parameters were set as *p*_*x*_ = 200 nm, *d* = 120 nm, *t*_1_ = 50 nm, and *t*_2_ = 50 nm. Comparing the three cases, the LSPR at 402 nm is relatively weak and the reflection electric field is strong, indicating a weak absorption of 82%. For the 502 and 680-nm wavelengths, the LSPRs are obviously stronger (shown in Fig. 6b, c), resulting in better absorptions of 99.4 and 99.6%, respectively. For a better understanding, the Poynting vectors are also plotted with electric field distributions. The greater magnitude of Poynting vector can be seen in the vicinity of nano-disk, especially for the cases with wavelengths of 502 and 680 nm. From the pattern of Poynting vector, a conclusion can be drawn that strong resonance around the nano-disk occurs and the energy flows into the nano-disk, i.e., the incident light energy is mainly absorbed by TiN nano-disks. Moreover, the enhanced field around the TiN nano-disks resulting from the LSPR improves the absorption in monolayer MoS_2_ which broadens the absorption band of TiN nano-disk/SiO_2_/Al structure for both the cases of monolayer MoS_2_ inserted upon and underneath the nano-disk array.

**Fig. 7:**
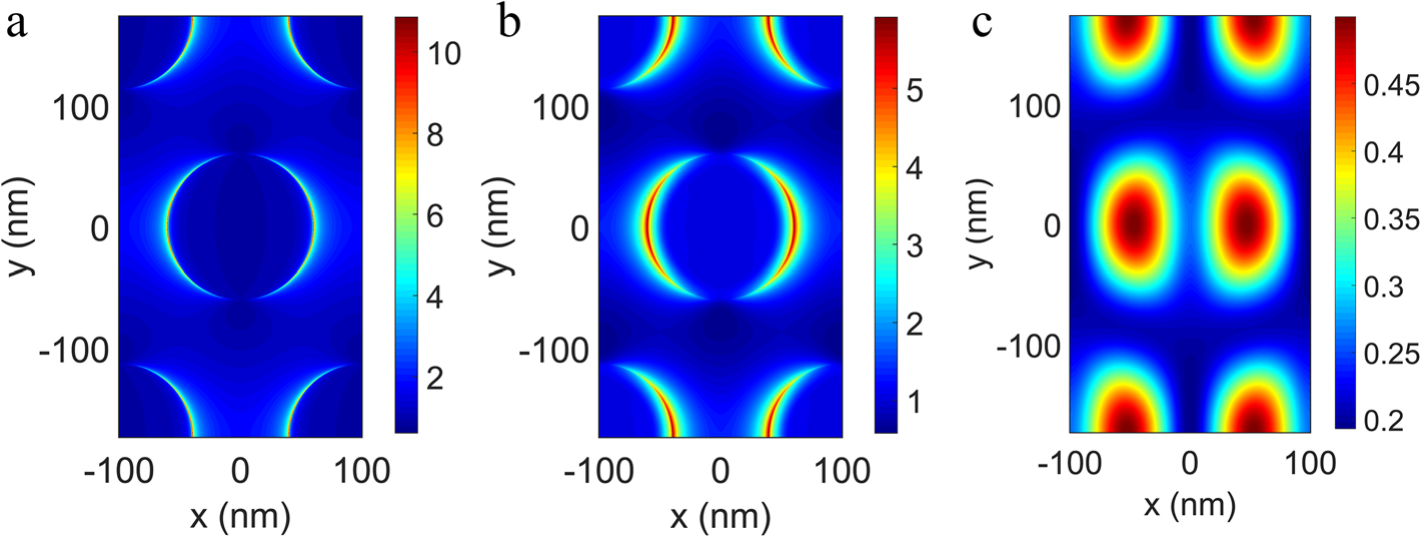
The electric field distribution in the *xy* plane for resonant wavelength of 668 nm on **a** interface 1: the top TiN disk surface, **b** interface 2: the interface between the TiN disk and SiO_2_ layer, and **c** interface 3: the interface between SiO_2_ layer and bottom TiN layer

To delve deeper, Figure 7a–c show field intensity distributions on the top surface of nano-disks (interface 1), the interface between the TiN nano-disks and SiO_2_ layer (interface 2), and the interface between the SiO_2_ layer and bottom Al substrate (interface 3) of a unit cell along the *xy* plane at the resonance wavelength 680 nm, respectively. All the intensity distributions are symmetric, and the maximum resonance intensity is at the edges of the TiN nano-disks indicating that oscillating charges accumulate there (Fig. 7a–c). Regarding interface 3, the resonance intensity is attenuated compared to that of interface 2 due to the scattering electromagnetic field by the nano-disks traveling through the SiO_2_ spacer layer along different directions. As Fig. 6d shows, the magnetic resonance is excited in the gap, which results in an artificial magnetic moment that interacts strongly with the magnetic field of the incident light [48]. Therefore, the electromagnetic field can be enhanced in the gap, and the energy is efficiently confined to the gap between TiN nano-disks and the Al substrate. When the LSPR and magnetic resonance are impedance-matched, the total absorption arrives at unity [19]. With increasing nano-disk diameter, the cross talks become stronger, leading to changing impedance-matching condition. As a result, one absorption peak splits into two absorption peaks at wavelengths 502 nm and 680 nm, where the LSPR and magnetic resonance are impedance-matched to unity absorption. In addition, the existence of magnetic resonance makes the monolayer MoS_2_ inserted underneath nano-disk array perform better than upon the nano-disk array. Above all, the broadband perfect absorption is obtained owing to excitation of the strong LSPR of nano-disks and magnetic resonance in the gap, along with the cross talks between the adjacent nano-disks. With the developed nanofabrication technology, our design can be realized by the process of thin film deposition and etching.

## Conclusion

In this work, a metamaterial perfect absorber in the waveband from 400 nm to 850 nm has been studied by the FDTD method. Our initially proposed TiN nano-disks/monolayer MoS_2_/SiO_2_/Al structure offers a broadband perfect absorption of average 98.1% from 400 nm to 850 nm, including an 300-nm bandwidth with near 100% (over 99%) absorption from 475 nm to 772 nm. The realization of the intriguing absorption is on account of the strong LSPR, cross talks of TiN nano-disks, and the magnetic resonance in the gap. Importantly, introducing a monolayer MoS_2_ have improved significantly the absorption performance. In addition, the perfect absorber shows polarization-insensitivity at normal incidence. In terms of compactness, the dimension of the metamaterial absorber can be reduced to 150 nm thick. In conclusion, the perfect absorber proposed in this work with the hexagonally periodic, circularly shaped TiN nano-disk pattern and monolayer MoS_2_ offers broadband near unity absorption and is promising for photovoltaic devices and light trapping.

## Abbreviations

FDTD: Finite-difference time-domain
LSPR: Localized surface plasmon resonance
MA: Metamaterial absorber
